# Spectrum and Immediate Outcome of Acute Kidney Injury in a Pediatric Intensive Care Unit: A Snapshot Study from Indian Subcontinent

**DOI:** 10.5005/jp-journals-10071-23217

**Published:** 2019-08

**Authors:** Ashwini Bharat, Anita Mehta, Harish Chandra Tiwari, Bhupendra Sharma, Abhishek Singh, Vijay Singh

**Affiliations:** 1 Department of Pediatrics, Christian Medical College, Vellore, Tamil Nadu, India; 2,4-6 Department of Pediatrics, BRD Medical College, Gorakhpur, Uttar Pradesh, India; 3 Department of SPM, BRD Medical College, Gorakhpur, Uttar Pradesh, India

**Keywords:** Acute kidney injury (AKI), Pediatric intensive care unit (PICU), pRIFLE, Viral encephalitis, Scrub typhus

## Abstract

**Background and aims:**

Acute kidney injury (AKI) became an important cause of mortality and morbidity in critically ill children, despite advancement in its management. In developing countries etiology of AKI are different from that of developed countries.

**Materials and methods:**

This observational study was carried out in pediatric intensive care unit (PICU) in 2 months to18 years of critically ill children. Kidney injury was defined and categorized by the pRIFLE criteria.

**Results:**

Out of 361children, 86 children (23.8%) developed AKI at some point during admission, 275 children (age and sex matched) who did not develop kidney injury during hospitalization served as non-AKI children. Maximum cases of AKI were seen in 1–5 years of age. Maximum children of AKI were of viral encephalitis (n = 43, 50.0%) followed by scrub typhus (n = 14, 16.3%). Risk factors for the development of AKI were shock, PRISM score and longer hospital stay. In our study the mortality in AKI children (n = 30, 34.8%) was significantly higher (*p* = 0.005) as compared to non-AKI children (n = 56, 20.3%)). Duration on mechanical ventilation, PICU stay and hospital stay were also significantly (*p* = 0.001) higher in AKI children.

**Conclusion:**

AKI is common in critically ill children and associated with high mortality and morbidity.

**How to cite this article:**

Bharat A, Mehta A, Tiwari HC, Sharma B, Singh A, Singh V. Spectrum and Immediate Outcome of Acute Kidney Injury in a Pediatric Intensive Care Unit: A Snapshot Study from Indian Subcontinent. Indian J Crit Care Med 2019;23(8):352-355.

## INTRODUCTION

Acute kidney injury (AKI) is a leading cause of morbidity and mortality in critically ill children. AKI is characterized by the abrupt deterioration of renal functional resulting from retention of endogenous or exogenous toxic metabolites which leads to reduction in glomerular filtration rate (GFR), rise of serum creatinine (SCr) and fluid, electrolyte and water imbalance.^1^ The incidence of AKI varies between 10% and 45% in different countries among different ethnic children.^2,3^ Despite advances in management of critically ill children, the mortality of AKI children is still high (30–40%) and a proportion of children may progress to chronic kidney disease (CKD). There are different methods for diagnosis and grading of AKI.^4^ pRIFLE criteria is commonly used for diagnosis, grading and prognosis of AKI.^5^

Burden and spectrum of AKI in developing countries is different from that of developed countries.^2,6^ In developed countries the underlying cause is predominantly postsurgical, malignancy, and nephrotoxic drugs and in developing countries, it is due to severe infection, diarrhea, hemolytic–uremic syndrome and glomerulonephritis.^5,7,8^ There is lack of literature of AKI in eastern UP, hence this study has been planned to know the burden, etiology and immediate outcome of AKI among hospitalized children in pediatric intensive care unit (PICU) of tertiary care hospital which would help in identifying the possible areas of intervention.

## MATERIALS AND METHODS

### Setting, Design and Duration of Study

**Study setting:** PICU, Department of Pediatrics, BRD Medical College, Gorakhpur, Uttar Pradesh.

**Study design:** Cross-sectional hospital-based study.

**Study duration:** July 2017 to October 2017.

### Sample Size

Sample size was calculated by using formula 4PQ/L2. The incidence of AKI varies between 10% and 45% in different countries among different ethnic children. Records at our centre reveals 30–40% incidence of AKI among children admitted in PICU. Calculated sample size was 336 by considering proportion of children admitted in PICU developing AKI as 30% (*p*=30%) with an allowable error (L) of 5%. We included 30% extra for non/incomplete responder/ deaths/other reasons for exclusion. Final sample size taken for screening was 436.

### Inclusion and Exclusion Criteria

All children admitted in PICU from 2 months to 18 years of age were enrolled for the study. Criteria for admission in PICU were need of mechanical ventilation, vasopressor support, having respiratory failure, fulminant hepatic failure and poorly controlled seizure. Post surgical children and children with oncological emergencies were not admitted in our PICU. Children who were less than 2 months of age, having CKD, PICU stay <24 hours and with major congenital anomaly were excluded from the study.

### Study Procedure

The study protocol was approved by the Institutional Ethics Committee. Well informed written consent in local language after explaining them the purpose of study was taken from mothers/guardians of each child. Children admitted in PICU fulfilling the inclusion criteria were enrolled. A detailed evaluation was done in all the children to ascertain the etiology, its progression and need for dialysis of AKI. PRISM III score was used for assessment of severity of illness during the first 24 hour of admission.^9^ All the children were managed according to standard management protocol. They were followed until discharge from PICU and then from hospital to determine recovery of renal function and need of renal replacement therapy (RRT). Indications for RRT were anuria or oliguria with shortness of breath, intractable metabolic acidosis, difficult to control hypertension, features of uremia, altered sensorium, severe hyperkalemia and recurrent vomiting in the setting of rise in serum creatinine.^10^

Serum creatinine and biochemical tests were done in departmental side lab. Serology for dengue, Japanese encephalitis (JE), scrub typhus, herpes virus and measles were done in Regional Research Centre of National Institute of Virology (NIV) Pune, situated in premises of our Institute. All the findings were recorded on a predefined working proforma.

Outcome measures in our study were need and duration of invasive mechanical ventilation, duration of stay both in the PICU and in hospital, and mortality.

### Assessment of Kidney Injury

Kidney injury was defined and categorized using the pRIFLE criteria.^5^ Maximum pRIFLE stage during PICU stay was recorded with special emphasis on time of onset and duration of kidney injury. Children, who did not develop AKI during stay at PICU, were taken as control. Baseline estimated CCl was calculated from serum creatinine measured within 3 months before PICU admission. In the absence of baseline creatinine, 100 mL/min/1.73 m^2^ was considered to be baseline eCCl. Serum creatinine level was estimated by auto analyzer by modified Jaffe method.^11^ It was done at admission and then daily, sometimes more frequently depending on patient's clinical condition. The CCl was estimated from the Schwartz formula.^12^

ECCl = K × Height (cm)/SCr

K: Constant, its value is 0.45 in <12 months, 0.55 in 1 and 12 years, 0.55 in females >12 years and 0.70 in males >12 years. eCCl more than 90 mL/min/1.73 m^2^ was considered normal. We classified AKI according to change in eCCl of children. Urine output criteria was not used in our study because it is affected by hydration state, use of diuretics, amount of intravenous fluid, and presence of obstruction.

### Statistical Analysis

SPSS version 22 (Armonk NY, IBM Company) was used for statistical analysis. Descriptive data were presented as percentages, means, and standard deviation. Chi-square test was used to study frequency distribution between children and when frequency was less than 5 Fisher's exact test was used. Independent sample ‘t’ test was used to compare means of AKI and non-AKI group. Binary logistic regression analysis has been done to find out risk ratio of important risk factors. *p* value <0.05 was considered significant.

## RESULTS

A total of 436 children were screened on admission, out of which 75 were excluded (12 CKD on admission, 3 congenital anomaly, 42 had <24 hour PICU stay and 18 refused to participate in study). Out of 361 who met the inclusion criteria, 86 children (23.8%) developed AKI at some point during admission, and of these 46 (53.4%) were reached as risk (R_max_), 24 (28%) as injury (I_max_), and 16 (18.6%) as failure (F_max_) grading. Two seventy five children (age and sex matched) who did not develop kidney injury during hospitalization served as non AKI children. Mean±SD PRISM III scores for non AKI, R_max_, I_max_, and F_max_ grading was 3.89±5.65, 6.3±2, 7.2±2.8 and 9.8±2.7 respectively.

The mean age of AKI and non-AKI children were 5.6 ± 3.4 and 5.9 ±3.2 years respectively. Male: Female ratio in AKI and non-AKI children was 1:1.3 and 1:1.1 respectively. Baseline characteristics of AKI and non-AKI children were comparable (Table 1). Nephrotoxic drugs used in our PICU were aminoglycosides, vancomycin and loop diuretics with a mean duration of 5 days.

Table 2 showing distribution of AKI and non-AKI children according to etiology. Maximum children in our PICU were of viral encephalitis (n = 145, 40.16%), followed by scrub typhus (n = 104, 28.8%). The children of viral encephalitis and scrub typhus together constituted more than half of total cases. Viral encephalitis included suspected and confirmed cases of dengue, JE, herpes and measles. Maximum children of AKI were also of viral encephalitis (*n* = 43, 50.0%) followed by scrub typhus (*n* = 14, 16.3%).

In Table 3, we estimated risk ratio of the risk factors responsible for AKI by logistics regression analysis. We found shock (RR 5.041; *p* = 0.014), PRISM III score (RR 0.392; *p* = 0.000) and hospital stay (RR 0.090; *p* = 0.000) as risk factors for the development of AKI in our patients.

**Table 1:** Baseline characteristics of AKI and non-AKI children

*Baseline characteristics*		*AKI* *n = 86 (±SD)*	*Non- AKI* *n = 275 (±SD)*	*p value*
Average age (years)		5.6±3.4	5.9 ±3.2	0.53
Average weight (kg)		15.4±3.6	17.4±6.5	0.14
Average height (cm)		106.5±3.4	108.2±6.2	0.12
Gender	Male	49 (56.9%)	144(52.3%)	0.45
	Female	37 (43.1%)	131 (47.7%)	0.45
Nephrotoxic drugs (n=)		42 (48.8%)	126 (45.8%)	0.62

**Table 2:** Distribution of AKI and non-AKI children according to etiology

*Etiology*	*AKI* *(n = 86, %)*	*Non-AKI* *(n = 275, %)*	*Total* *(n = 361, %)*
Viral encephalitis	43(50%)	102 (37%)	145 (40.2%)
Scrub typhus	14 (16.3%)	90 (32.8%)	104 (28.8%)
Sepsis	12(14%)	13(4.7%)	25 (6.93%)
Gastroenteritis	10 (11.7%)	8 (3%)	18 (4.99%)
Diabetes mellitus	2 (2.3%)	24(8.8%)	26 (7.20%)
Tubercular meningitis	3 (3.4%)	21(7.6%)	24 (6.65%)
Hepatic encephalopathy	2 (2.3%)	17(6.1%)	19 (5.26%)
Total	86 (100%)	275(100%)	361

**Table 3:** Multivariate analysis of AKI and non-AKI children

*Variables*	*AKI* *n = 86 (%)*	*Non AKI* *n = 275 (%)*	*P value*	*RR*
Shock	48 (55.8%)	68 (24.7%)	0.014	5.041
PRISM III score	7.34±2.79	3.89±5.65	0.000	0.392
Hospital stay (days)	7.7 ±2.3	5.5 ±1.6	0.000	0.090

**Table 4:** Outcome of AKI and non-AKI children

*Variables*	*AKI n = 86 (%)*	*Non-AKI n = 275 (%)*	*p value*
Mortality	30 (34.8%)	56 (20.3%)	0.005
Duration of mechanical ventilation (days)	5.2 ±1.6	3.2 ±1.2	0.001
Duration of PICU stay (days)	6.3 ±1.9	4.5 ±1.4	0.001
Duration of hospital stay (days)	7.7 ±2.3	5.5 ±1.6	0.001

**Table 5:** Outcome and grading of AKI

*Variables*	*Risk* *(n = 46, 53.5%)*	*Injury* *(n = 24, 27.9%)*	*Failure* *(n = 16, 18.6%)*	*p value*
Mechanical ventilated (n = 43)	13 (28%)	17(70%)	13(81%)	
Duration of ventilation (days)	4.3±0.59	5.2±1.3	5.2±1.8	0.0002
PICU stay (days)	5.5±2	6.7±2	8±2	0.0001
Hospital stay (days)	6.8±1.8	7.5±1.6	9±1.7	0.0004

We found 33.6% (*n*=29) children developed AKI within 24 hours of hospitalization. Maximum cases (*n*=54, 63%) developed AKI between 1 day and 7 days and only 3 (3.4%) children developed after 7 days. All of the children developed AKI during PICU hospitalization. RRT was required in 11 (16.1%) children. Two children of R_max_ (4.35%), 4 of I_max_ (16.67%) and 5 of F_max_ (25.0%) grading required RRT.

In our study (Table 4) the mortality in AKI children (n = 30, 34.8%) was significantly higher (*p* = 0.005) as compared to non AKI children (*n* = 56, 20.3%)). The duration on mechanical ventilation, PICU stay and hospital stay were also significantly (*p* = 0.001) higher in AKI children than in non-AKI.

In Table 5, outcome measures were compared with AKI grading. There is statistically significant difference for hospital stay (*p* = 0.0004), PICU stay (*p* = 0.0001) and for duration on mechanical ventilation (*p* = 0.0002) with severity of AKI grading.

## DISCUSSION

This study was carried out in a tertiary care hospital to know the spectrum, etiology and immediate outcome of AKI among hospitalized children in PICU from 2 month to 18 years of age during a specified period of tine i.e. from July to October. The incidence of AKI in our PICU children was 23.8%. The incidence of AKI in different studies varied between 6.2% and 42.9%.^13–21^ This varied incidence may be because of different ethnic group, different inclusion criteria for study, different admission criteria in PICU, varied etiology of critically ill children, varied risk factors and inconsistent use of AKI classification criteria. Some authors had used AKIN criteria and others pRIFLE criteria. In pRIFLE criteria also some had used both eCCl and urine output criteria and others only eCCl criteria.

Our study showed a very distinct distribution of disease etiology in both AKI and non-AKI group. Viral encephalitis and scrub typhus was the etiology in majority of cases in our study. Majority of authors reported sepsis as a leading cause of AKI in their study.^13,15–17,21^ Others had reported pneumonia as a commonest etiology of AKI.^19,20^ In few studies hemato-oncologic diseases and postoperative cases were the commonest etiologies.^18,20^ These differences could be due to regional differences in study populations, varied disease etiology, difference in demographic profile of critical disease, different admission criteria and admission policy in PICU. Eastern Utter Pradesh is high endemic area for viral encephalitis particularly during July to November months, therefore maximum cases in our study were of viral encephalitis.^23^ In last few years scrub typhus became a leading cause of encephalitis in this region.^24^ Scrub typhus was also reported as an important cause of AKI from different parts of India also.^25–28^ In our study within 24 hours and within 7 days of admission, AKI developed in 33.6% and 96.5% of children respectively. Other authors had also reported maximum children in their study developed AKI within 72 hours of admission.^14,18,19,21,22^

We studied the risk factors for the development of AKI in PICU children. Shock, high PRISM III score and longer duration of hospital stay were associated with development of AKI in our study. Similar results were reported by other authors also.^14,19,20^

In present study AKI cases reached into R_max_, I_max_ and F_max_ grading at some point of time during hospitalization in 53.4%, 28% and 18.6% cases respectively. Other researchers also reported similar results.^5,14–16,19,21^ But in our study R_max_ cases were more than three times of F_max_ cases, as seen by other author also.^15^

In our study the children who developed AKI during hospitalization had higher (34.8%) mortality as compared to non AKI group (20.3%) and this difference was statistically significant (*p*= 0.005). The duration on mechanical ventilation, PICU stay and hospital stay were also significantly (*p*=0.001) higher in AKI children than in non-AKI. Similar results were reported by other authors also.^14,18,19,21,29^

## CONCLUSION

AKI is common in critically ill children and associated with high mortality and morbidity. It is associated with longer PICU and hospital stay, leading to a major burden on the healthcare system.
